# Microbial Symbionts Accelerate Wound Healing via the Neuropeptide Hormone Oxytocin

**DOI:** 10.1371/journal.pone.0078898

**Published:** 2013-10-30

**Authors:** Theofilos Poutahidis, Sean M. Kearney, Tatiana Levkovich, Peimin Qi, Bernard J. Varian, Jessica R. Lakritz, Yassin M. Ibrahim, Antonis Chatzigiagkos, Eric J. Alm, Susan E. Erdman

**Affiliations:** 1 Division of Comparative Medicine, Massachusetts Institute of Technology, Cambridge, Massachusetts, United States of America; 2 Laboratory of Pathology - Faculty of Veterinary Medicine, Aristotle University of Thessaloniki, Thessaloniki, Greece; 3 Biological Engineering, Massachusetts Institute of Technology, Cambridge, Massachusetts, United States of America; 4 Broad Institute of Massachusetts Institute of Technology and Harvard, Cambridge, Massachusetts, United States of America; Sapienza University of Rome, Italy

## Abstract

Wound healing capability is inextricably linked with diverse aspects of physical fitness ranging from recovery after minor injuries and surgery to diabetes and some types of cancer. Impact of the microbiome upon the mammalian wound healing process is poorly understood. We discover that supplementing the gut microbiome with lactic acid microbes in drinking water accelerates the wound-healing process to occur in half the time required for matched control animals. Further, we find that *Lactobacillus reuteri* enhances wound-healing properties through up-regulation of the neuropeptide hormone oxytocin, a factor integral in social bonding and reproduction, by a vagus nerve-mediated pathway. Bacteria-triggered oxytocin serves to activate host CD4+Foxp3+CD25+ immune T regulatory cells conveying transplantable wound healing capacity to naive Rag2-deficient animals. This study determined oxytocin to be a novel component of a multi-directional gut microbe-brain-immune axis, with wound-healing capability as a previously unrecognized output of this axis. We also provide experimental evidence to support long-standing medical traditions associating diet, social practices, and the immune system with efficient recovery after injury, sustained good health, and longevity.

## Introduction

The great importance of gut microbiota in mammalian host health is only recently being recognized in full [1]–[4]. There is now substantial evidence linking naturally-occurring or experimentally-induced disturbances of the intestinal microbial communities with immune-associated and metabolic diseases, and with neoplasia [2]–[3], [5]–[7]. Accordingly, modifying the gut microbiota using dietary probiotics is now accepted as an emerging therapeutic and overall health promoting treatment [1], [3]–[4], [8]. Nonetheless, the mechanisms by which gut microbiota impart effects that expand beyond the gastrointestinal tract and become systemic are largely elusive. The most compelling data explaining such phenomena come from studies demonstrating that gut bacterial organisms and their metabolites interact with interrelated immunological [9]–[12], metabolic [2]–[4], [13] and neuroendocrine pathways [13]–[15].

During a recent study we observed that female mice fed with the lactic acid bacterium *Lactobacillus reuteri* (*L. reuteri)* show more frequent grooming activity compared to their control counterparts [16]. This aspect of maternal behavior is regulated at large by the neurohypophyseal hormone oxytocin, best known for its role in parturition and lactation. Our understanding of oxytocin’s classical role, however, has greatly expanded over the last decades to include substantial central nervous system effects on behavior [17]–[19]. Interestingly, its most recently discovered but not as well-characterized roles include interactions with body energy balance [20]–[21] and the immune system [22]–[24].

The hypothesis that the ingestion of a lactic acid bacterium could up-regulate the expression of the neuropeptide hormone oxytocin in the hypothalamus, although without precedent, has substantive indirect support. Indeed, considerable evidence suggests the presence of a “microbiome-gut-brain axis” [14]–[15], [25]–[26]. According to this reasoning, bacteria, including ‘probiotic’ organisms, initiate immune-related and neural signals that are transmitted from the gut to the CNS, either through blood circulation or directly via the vagus nerve [15], [25]–[28] [29]. This crosstalk between the gut microbiota and vital regulatory components of the CNS, such as the hypothalamus and pituitary gland is thought to impact mammalian homeostasis, including both physical and mental health [2]–[4], [13], [15].

In the present study, we find that administering purified *L. reuteri* organisms in drinking water induces a significant up-regulation of the neuropeptide hormone oxytocin in mice. In order to probe downstream effects of this microbial mode of oxytocin up-regulation within a tractable mammalian model system, we used a well-characterized experimental model of cutaneous wound healing in mice [30]. We selected this approach primarily because our earlier cancer studies revealed unusually robust integumentary health in aged animals when they consumed purified *L. reuteri* [16]. In these studies, the mouse models consuming *L. reuteri* exhibited mucocutaneous hyperacidity, follicular anagenesis, and sebocytogenesis mimicking features of superb physical fitness in a gender-dependent manner.

Additionally, wound healing is a fundamental process with mechanisms inextricably linked with health and disease, such as hemostasis, fibrosis, tissue repair and remodeling, acute and chronic inflammation, and neoplastic tumor evolution [31]–[33]. Our findings suggest that *L. reuteri* impacts the host animal inflammatory response in an oxytocin-mediated fashion. The oxytocin-potent acceleration of wound healing benefit depends upon vagus nerve integrity and is transplantable using either CD4^+^Foxp3^+^ or CD4^+^CD45RB^lo^CD25^+^ T regulatory immune cells (Tregs) to completely recapitulate the *L. reuteri*-induced phenomenon in Rag2-deficient recipient host animals. These findings reveal unexpected roles of oxytocin as a novel component of the gut microbiome-associated biological networks linking mental, social, and physical health, with widespread potential benefits for high quality and healthful life.

## Results

### Consumption of *Lactobacillus reuteri* compresses the classical wound repair cascade

**Figure 1:**
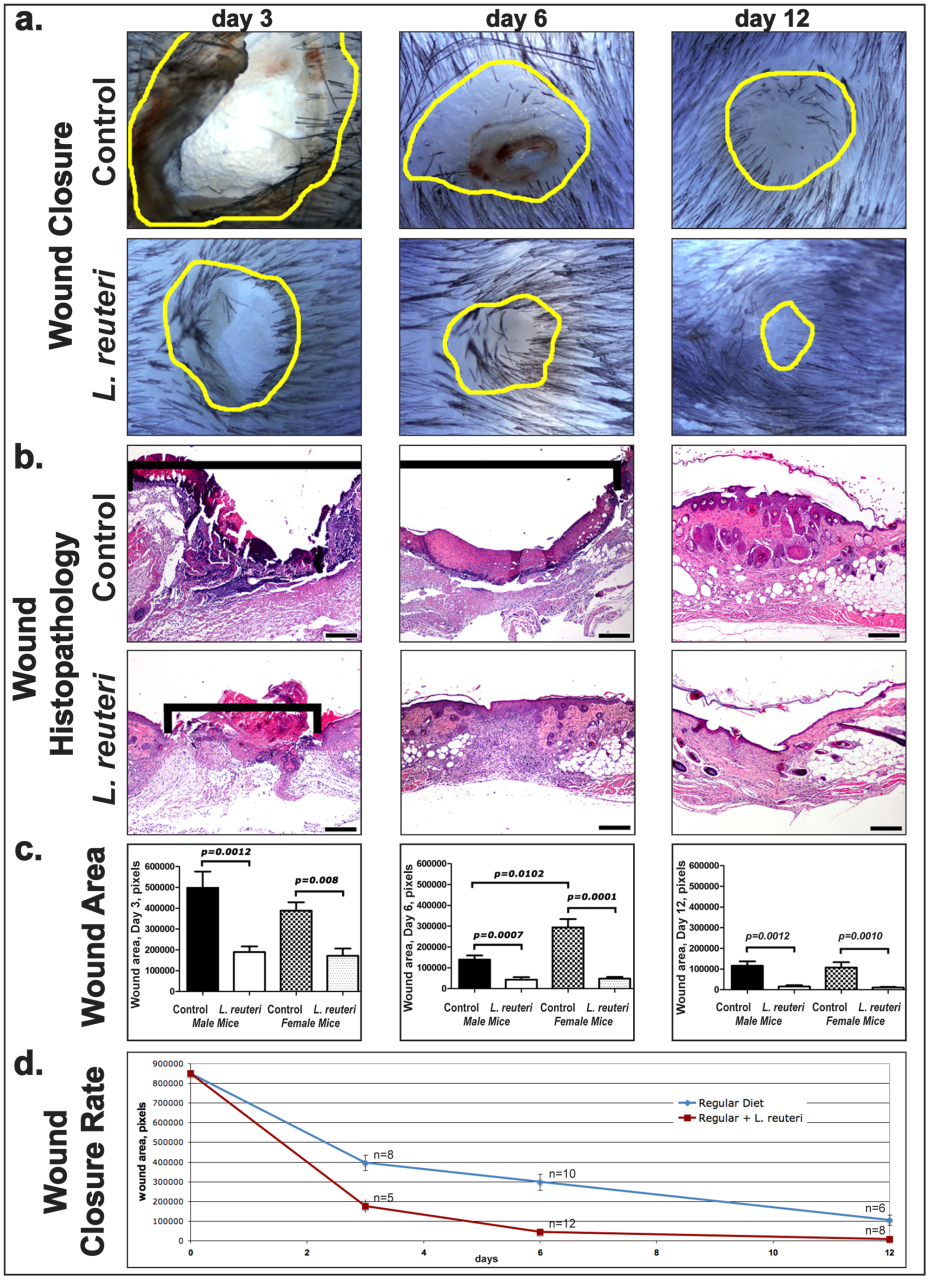
Dietary supplementation with *L. reuteri* accelerates wound healing. (a) Microscopy of formalin-fixed, paraffinized wounded skin of aged C57BL/6 mice (at 3, 6, and 12 days post- wounding). Wound margins are delineated with yellow outlines. The healing time course is faster in mice consuming *L. reuteri* evidenced by reduced wound sizes. (b) Histopathology of wound healing timecourse shows wound epidermal gaps [indicated by black brackets]. Accelerated epidermal closure in mice consuming the purified lactic acid bacteria led to complete re-epithelialization of wounds in 8/12 female mice by the 6th day post-wounding. By contrast, zero of 12 control animals had complete epidermal wound closure at the same time-point. 12 days after biopsy, the newly formed epidermis in *L. reuteri*-treated mice was normal and lacked regenerative hyperplasia, indicating a rapid rate of epithelial remodeling. (c) Wound area at each of three time points decreases significantly in both male and female mice fed *L. reuteri*. Female mice fed *L. reuteri* exhibit more significant wound closure compared to controls versus male mice at 6 and 12 days. (d) Wound size diminishes more rapidly in mice fed *L. reuteri* with the increased rate of wound closure, accompanying a smaller epidermal gap in both male and female mice. Hematoxylin and Eosin. Scale bars: a =  250 µm. (3 day: Male: Control (n = 6), Control + LR (n = 7), Female: CD (n = 6), CD+LR (n = 8); 6 day: Male: Control (n = 12), Control + LR (n = 12); Female: Control (n = 12), Control + LR (n = 12); 12 day: Male: Control (n = 7), Control + LR (n = 7), Female: Control (n = 9), Control+LR (n = 8)).

**Figure 2:**
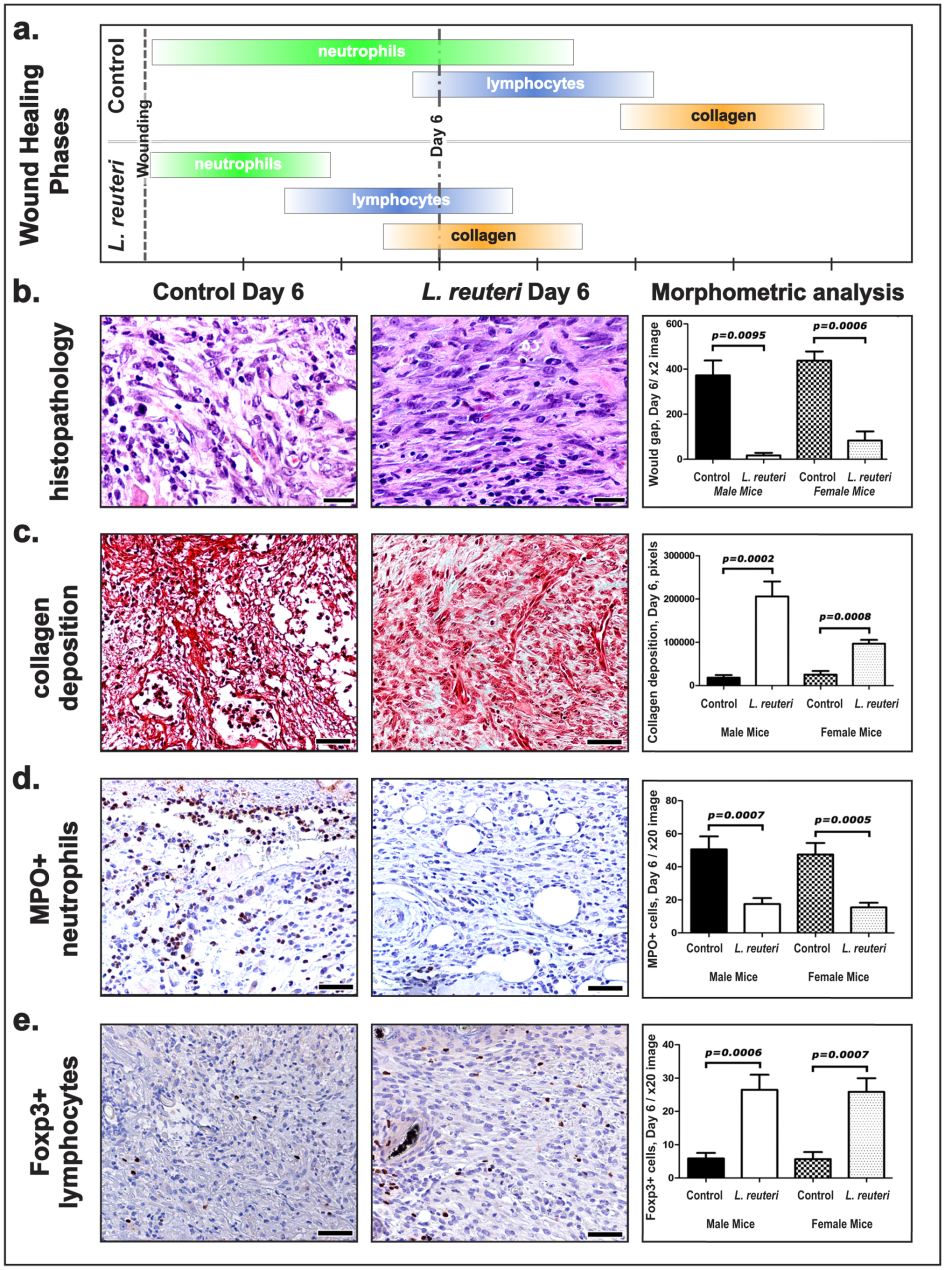
Immune cell profile of wounds in *L. reuteri*-treated mice differ from untreated counterparts. (a) Compression of the wound healing cascade in *L. reuteri*-treated mice results in neutrophil departure by day 6 and the beginning of collagen deposition showing advanced healing. Control animals wounds' remain populated with neutrophils and other innate immune cell infiltrates, indicative of a comparatively early stage of wound healing. (b) Histopathology of the granulation tissue in wounds of male mice. Early granulation tissue in control mice characterized by activated plump fibroblasts, (c) minimal amount of collagen, and (d) abundant neutrophils with (e) small numbers of Treg cells. The granulation tissue of mice fed *L. reuteri* is more mature with (b) elongated fibroblasts and a chronic inflammatory component (lymphocytes), (c) increased collagen deposition, and (d) small numbers of neutrophils and (e) abundant Treg cells. (6 day: Male: Control (n = 12), Control + LR (n = 12); Female: Control (n = 12), Control + LR (n = 12)). (b) Hematoxylin and Eosin. (b) Masson's Trichrome. (d) and (e) Immunohistochemistry: Diaminobenzidine chromogen, Hematoxylin counterstain. Scale bars = 50 µm.

Wound healing capability is the hallmark of sustained good health [31]. During our carcinogenesis studies, we discovered dramatically improved skin and mucosal health in animal subjects consuming probiotic-containing yogurt [16]. We hypothesized that microbes in the yogurt led to this improved epithelial phenotype. We tested our hypothesis using animal models consuming a purified preparation of lactic acid bacteria *Lactobacillus reuteri* ATCC-PTA-6475 [16], [34] suspended in their regular drinking water. To specifically assay wound healing capability, we first employed a traditional skin biopsy assay in aged six-month-old C57BL/6 *wild type* (*wt*) mice to quantify the extent to which consuming these organisms may impact wound healing. This assay applies a standardized 2.0 millimeter full thickness excision of dorsal skin [35]. Using this approach and then microscopically examining the excision site of these mice at three, six and twelve days after biopsy (Figures 1a and 1b), we found that aged mice of either gender consuming *L. reuteri* exhibited proper injury repair in half the time needed for control mice drinking regular water (Figs. 1c and 1d, and Fig. 2a). By measure of wound area, female mice consuming *L. reuteri* experienced a more marked increase in healing compared to their controls over male counterparts (Fig. 1c), but both sexes demonstrated similar improvements. This rapid wound-healing attribute of *L. reuteri* was not a generic feature of consuming any bacterium, as similar dosage levels of another microbe *Escherichia coli* strain K12 in drinking water did not significantly impact the wound healing process. (Wound closure at day 6: E.coli-treated (n = 5) vs Control (n = 5), p = 0.5476; E.coli-treated vs *L. reuteri*-treated (n = 5), p = 0.0079).

### Ingestion of lactic acid bacteria hastens collagen deposition

Rapid collagen deposition is a key feature of proper wound repair [36]. Using *in situ* labeling methods and histomorphometry we found complete epidermal closure and scab detachment by the sixth day post-wounding in *wt* mice consuming *L. reuteri* (Fig. 2b) with significantly accelerated maturation of the granulation tissue and collagen deposition in the dermis occurring by day 6 post-biopsy in mice drinking the *L. reuteri* suspension (Fig. 2c, Fig. S1). Granulation tissue of *L. reuteri*-treated mouse wounds exhibited activated fibroblasts, layers of quiescent elongated fibroblasts, and a denser extracellular matrix. By contrast, excision biopsy wounds of control mice drinking regular water at day 6 had only early stage granulation tissue involving edematous loose connective tissue elements, with small numbers of activated (plump) fibroblasts and minimal fibrosis (Fig. 2c, Fig. S1). Indices of cellular proliferation (Fig. S2) and apoptosis (Fig. S3) of epithelial cells, neutrophils and fibroblasts at the same time-point further elucidate the more rapid unfolding but non-pathogenic wound healing process exhibited in animals eating *L. reuteri*.

### Lactic acid bacteria confer more rapid progression of inflammatory events during wound healing

Differences in granulation tissue architecture also coincided with different inflammatory cell components, as evidenced by microscopic immunohistochemically-labeled cell count comparisons within wounds at six days after biopsy (Fig. 2a, Figs. 2c-e). Mice consuming *L. reuteri* displayed significantly reduced myeloperoxidase (MPO)-positive neutrophils (Fig. 2d) within their convalescent wounds, when compared with untreated control mice. *L. reuteri-*treated mice exhibited particularly large numbers of wound-associated Foxp3+ cells (Fig. 2e). Dietary intake of lactic acid bacteria has been shown elsewhere to induce potent Foxp3+ regulatory T (Treg) lymphocytes that down-regulate host inflammatory responses and serve to minimize collateral tissue injury [37].

**Figure 3:**
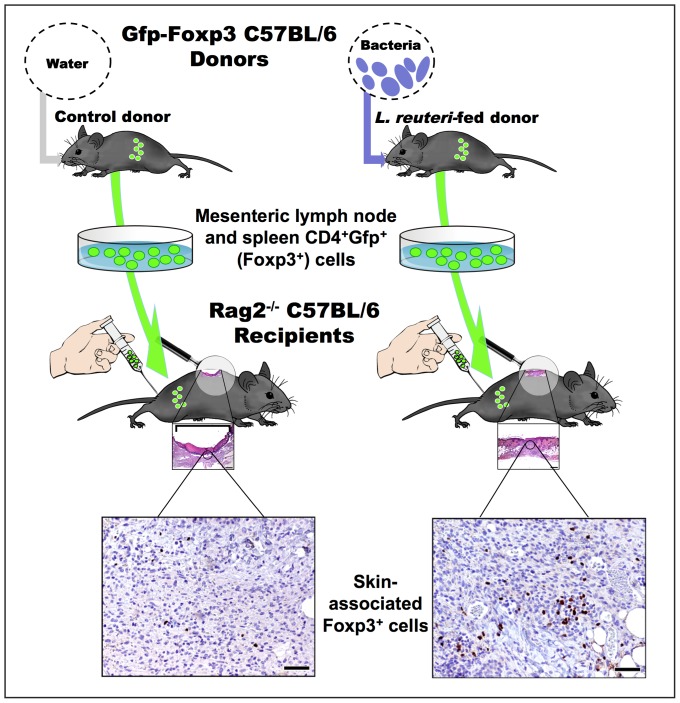
Adoptive cell transfer of Foxp3+ Tregs. Spleen and mesenteric lymph nodes from C57BL/6 donor mice are sorted for CD4+GFP+ (Foxp3+) regulatory T cells. Collected cells are transferred to lymphocyte-naive Rag2–/– C57BL/6 recipients. Transferred Foxp3+ cells migrate to the skin, where they act during wound healing.

**Figure 4:**
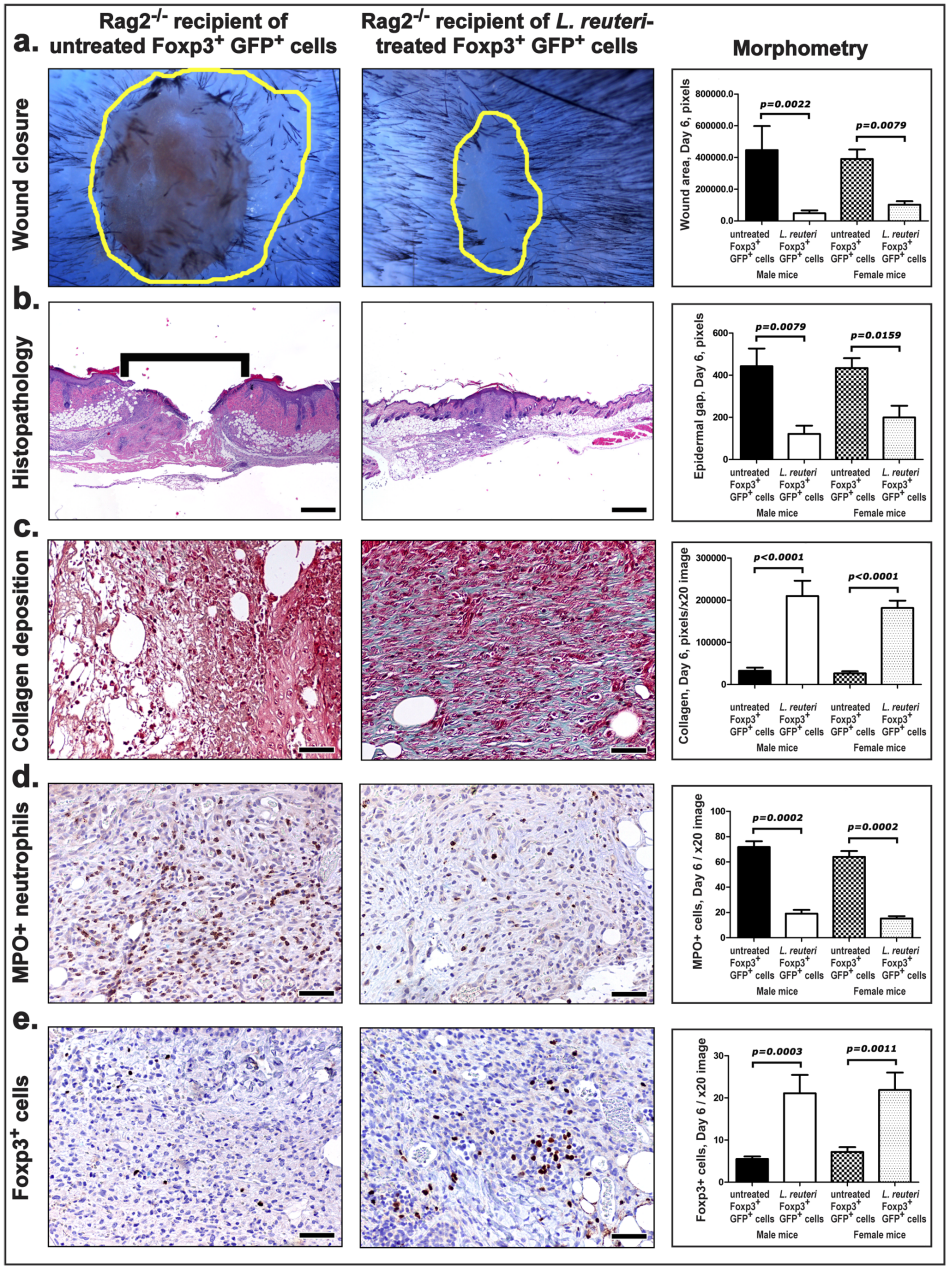
*L. reuteri*-primed Foxp3+ Tregs condense wound healing time course in Rag2–/– recipients. (a) Direct microscopy of formalin-fixed, paraffinized wounded skin of aged Rag2–/– C57BL/6 mice (6 days post-wounding). Wound margins are delineated with yellow outlines. The healing time course is faster in mice receiving *L. reuteri*-primed Foxp3+ Tregs as evidenced by significantly reduced wound sizes in both male and female recipient mice. (b) Significantly advanced re-epithelialization of wounds of Rag2–/– mice after adoptive cell transfer of Foxp3+ cells from *L. reuteri*-treated donors. (c) Early granulation tissue in mice receiving Foxp3+ Tregs from untreated controls is characterized by minimal amount of collagen, and (d) abundant neutrophils with (e) small numbers of Foxp3+ lymphocytes. The granulation tissue of mice receiving *L. reuteri*-primed Tregs is more mature with (c) increased collagen deposition, and (d) occasional neutrophils and (e) increased accumulation of the transferred Foxp3+ cells lymphocytes. (LR Foxp3+GFP+ cells (n = 6), Untreated Foxp3+GFP+ cells (n = 5) for each gender.) b) Hematoxylin and Eosin. (b) Masson's Trichrome. (d) and (e) Immunohistochemistry: Diaminobenzidine chromogen, Hematoxylin counterstain. Scale bars (b) = 250 µm; (c), (d) and (e) = 50 µm.

To test whether lactic acid bacteria-stimulated Foxp3+ Treg cells are important mediators in the accelerated wound healing process, we next applied a well-established adoptive transfer paradigm using highly purified CD4^+^Foxp3^+^ Treg cells. Lymphocytes were collected from twelve-week-old "Foxp3^EGFP^" C57BL/6 transgenic mice, which co-express eGFP and the regulatory T cell-specific transcription factor Foxp3. Donor mice were consuming *L. reuteri* or untreated drinking regular water for 3–4 weeks prior to cell harvest (Fig. 3) [38]–[40]. To test impact of CD4+Foxp3+ Tregs on host physiology, CD4+ cells were collected from mesenteric lymph nodes and spleens of Foxp3^EGFP^ mice, then sorted by flow cytometry for GFP expression, and finally 4X10^3^ count of CD4^+^GFP^+^Foxp3^+^ Tregs were injected intraperitoneally into C57BL/6 strain *Rag2*-deficient (*Rag2-KO*) animals otherwise absent functional lymphocytes (Fig. 3) [39], [41]. This transplantation of CD4^+^Foxp3^+^ Tregs was sufficient to recapitulate the accelerated wound closure (Fig. 4a), histology (Fig. 4b), collagen deposition (Fig. 4c), and immune cell infiltrates (Fig. 4d) in Rag2-KO cell recipients examined at six days after excision biopsy. Importantly, CD4^+^Foxp3^+^ Tregs collected from *L. reuteri*-fed mice were significantly increased within the wounded skin sites of Rag2-KO recipient mice (Fig. 4e). These data showed that CD4+Foxp3+ Treg cells were sufficient to convey the *L. reuteri*-imbued rapidity of wound closure after excision biopsy.

### Lactic acid microbe-triggered benefits require host ability to mount immune tolerance

**Figure 5:**
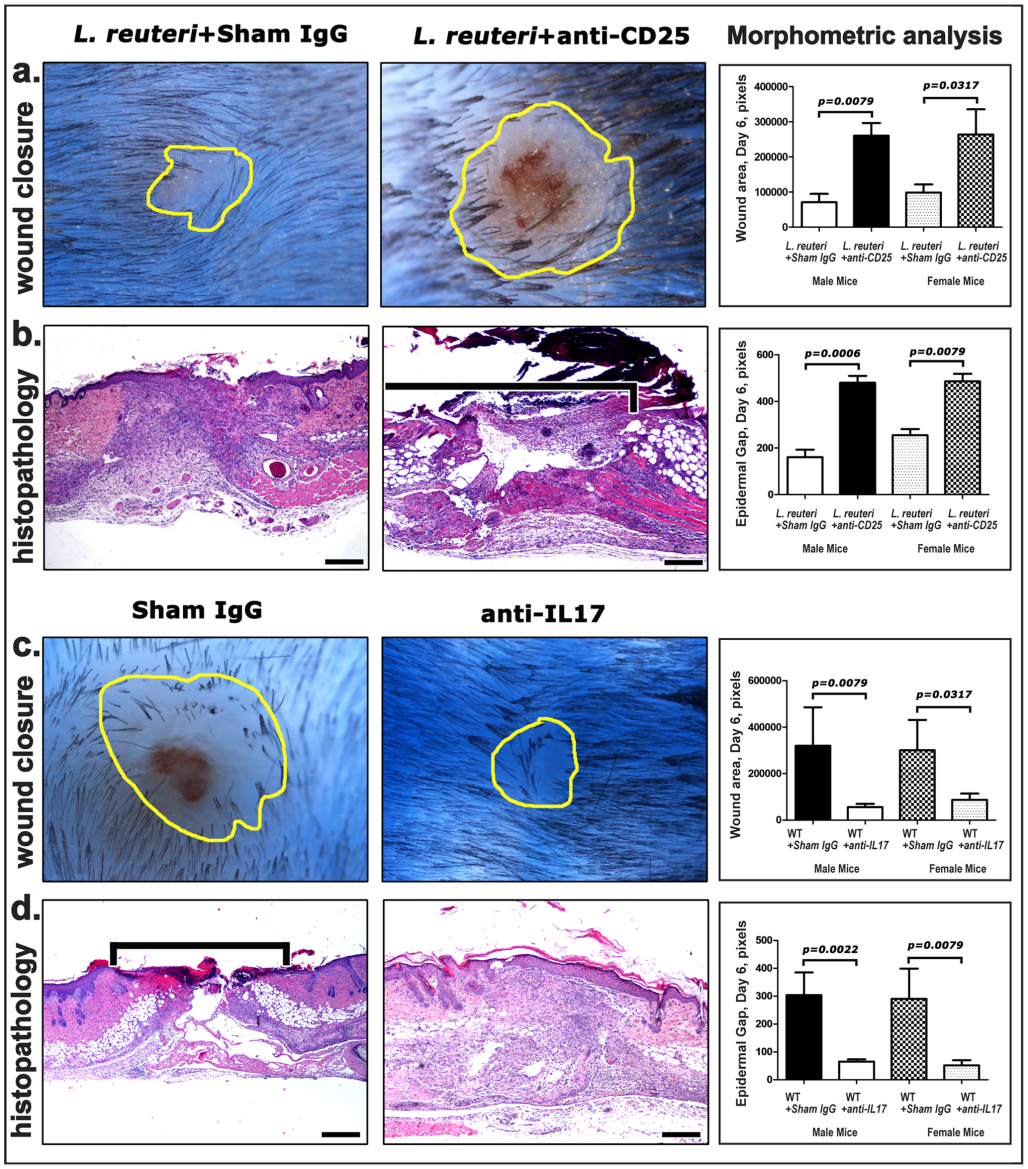
Depletion of CD25+ cells abolishes the *L. reuteri* effect in wound healing while depletion of IL-17 restores the wound healing benefit. (a) Male and female C57BL/6 mice (n = 8 per group) depleted of CD25+ cells by anti-CD25 antibody have larger wounds when compared with sham isotype IgG-treated control mice, despite uniform *L. reuteri* consumption in both groups. (b) The wounds of CD25 cell-depleted mice do not show the histopathological evidence of the typical *L. reuteri*-induced accelerated wound repair process, namely sham IgG exhibit complete epidermal closure and mature granulation tissue filling of the wound gap at 6 days after biopsy. (c) (d) Depletion of IL-17A benefits wound healing closure. Hematoxylin and Eosin (b and d). Scale bars =  250 µm.

Knowing that CD4^+^Foxp3^+^CD25^+^ Tregs bestow immune tolerance and homeostasis via down-regulation of inappropriate host inflammatory responses [37], [42]–[43], we next examined requirements for CD25^+^ cells which embody immune tolerance in the wound repair process. Using CD25 depletion studies via intraperitoneal injections of anti-CD25 or sham isotype antibody, we found that *L. reuteri*-induced improvements in C57BL/6 wt mouse skin wound closure required CD25^+^ cells (Figs. 5a and 5b), supporting a critical role for antigen educated CD25^+^ immune cells in the wound healing process. A well-known target of CD4^+^CD25^+^ Treg anti-inflammatory activity is the pro-inflammatory cytokine Interleukin (IL)-17A [44]–[45]. We subsequently tested whether inflammation in the form of IL-17A was responsible for delaying the wound healing process. As predicted, the experimental depletion of IL-17A using neutralizing antibodies in C57BL/6 *wt* mice contributed to significantly more rapid wound closure (Figs. 5c and 5d). Taken together, we propose that *L. reuteri*-induced CD4^+^Foxp3^+^CD25^+^ Tregs serve to down-regulate excessive inflammation, and in this way subsequently expedite the wound repair process.

During these studies, we observed that the rapidly healing C57BL/6 mice receiving the *L. reuteri* supplementation also displayed increased grooming behaviors, matching our earlier published observations [16]. The neuropeptide hormone oxytocin has been implicated in increased mother-infant grooming [46], and has also been linked with immune health [24] and improved wound healing [47]. This led us to postulate a unifying role for oxytocin linking lactation with microbes such as *L. reuteri* in more efficient injury repair culminating in improved host fitness.

### Plasma oxytocin levels increase after consuming *Lactobacillus reuteri*

Noting the requirement for oxytocin in normal mammalian wound healing processes [22]–[24], we hypothesized that the immune modulation conferred by *L. reuteri* may originate in the hypothalamic tract. Indeed, mice fed *L. reuteri* exhibited increased grooming [16], an activity regulated by the neurohypophyseal hormone oxytocin featured in infant-mother bonding [46]. Because oxytocin has previously been implicated in wound healing [47] and immune health [24], we postulated that oxytocin may serve to bridge the bacteria-triggered behaviors and physical fitness. We tested plasma oxytocin levels in our female C57BL/6 *wt* mice and found significant systemic elevation of this hormone in animals drinking *L. reuteri* daily when compared with matched untreated controls. (Control (n = 13): 380.5±46.82 pg/ml vs *L. reuteri* (n = 11): 875.3±141.7, p = 0.0004.)

Finding that oral *L. reuteri* therapy increased circulating levels of oxytocin in our animal model, we theorized that microbes associated with milk consumption may stimulate vagal pathways as for pleasure reward during infant-maternal bonding [29]. Indeed, it was recently shown that favorable mood after consuming other *Lactobacillus sps* was abolished after surgical transection of vagal nerve connections to viscera [26]. To test this possibility we performed vagotomies, and found that C57BL/6 *wt* mice undergoing vagotomy lost the *L. reuteri*–induced oxytocin surge phenomenon (Fig. S4a). At the same time, skin wound closure was delayed after vagotomy surgery in *wt* mice consuming *L. reuteri,* when compared with *L. reuteri*-treated animals undergoing sham surgery (Fig. S4b and S4c). Knowing that oxytocin has been implicated in wound healing [47] and immune health [24], we went on to test roles for oxytocin during *L. reuteri-*induced wound repair events in our mouse models.

### Oxytocin is required for the *L. reuteri*-induced boost in injury repair

**Figure 6:**
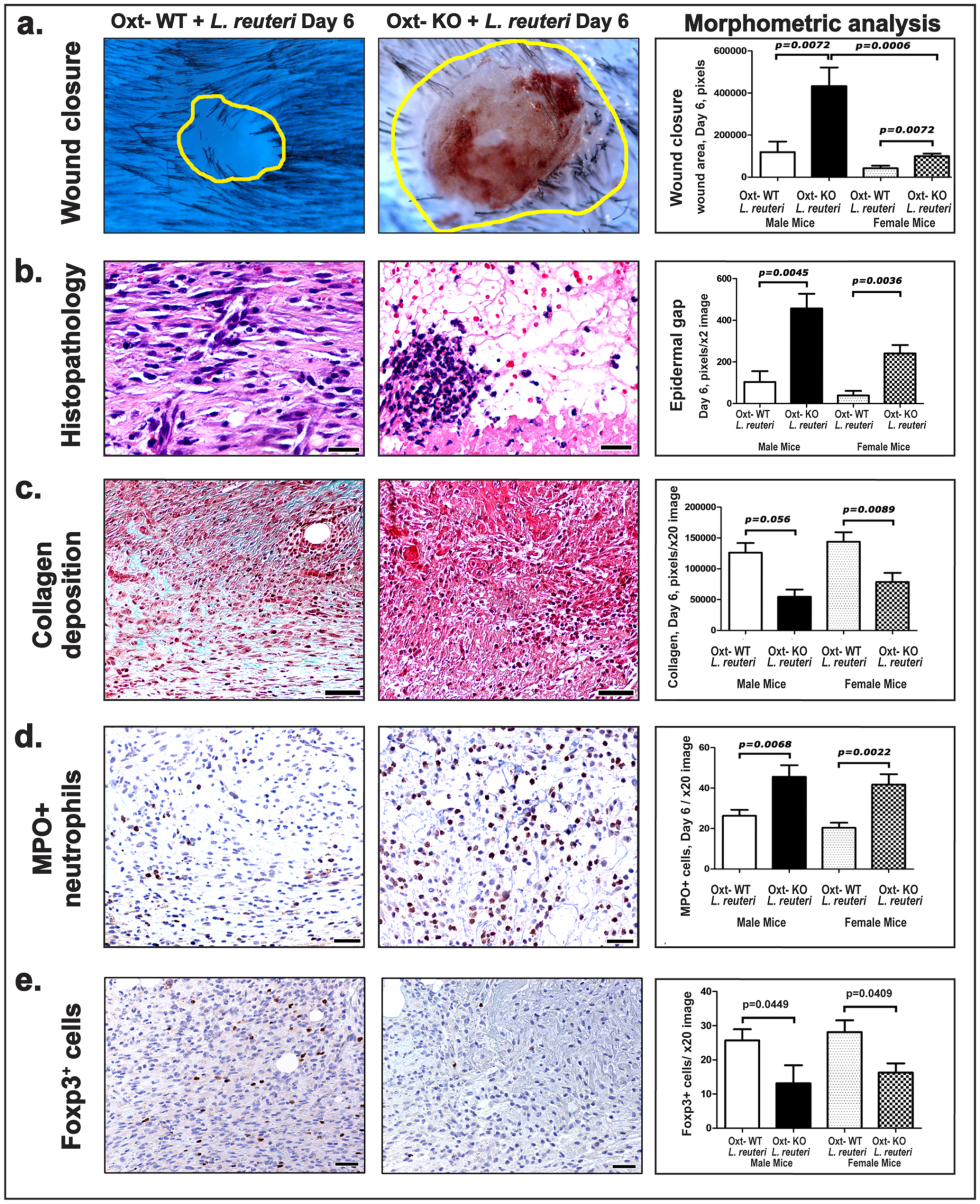
*L. reuteri* accelerates wound repair via an oxytocin-associated mechanism in both male and female mice. (a) Oxytocin-deficient male mice have significantly larger wounds in day 6 after wounding compared to wild-type control mice despite consumption of *L. reuteri*. Wound margins are delineated with yellow outlines. (b) Impaired wound healing in oxytocin-deficient mice is characterized by delayed re-epithelialization and granulation tissue formation. The mature granulation tissue of control mice is characterized by fibrosis and vessels running perpendicularly to the layers of elongated fibroblasts. In oxytocin-deficient mice there is still edematous (early) granulation tissue with an acute inflammatory component. (c) Oxytocin-deficient mouse wounds show minimal collagen deposition and significantly more (d) neutrophils. (b) Hematoxylin and Eosin. (c) Masson's Trichrome (d). Immunohistochemistry (Diaminobenzidine chromogen, Hematoxylin counterstain). Scale bars (b) = 25 µm; (c) (d) and (e)  = 50 µm.

To test whether oxytocin is necessary for *L. reuteri* -induced improvement in wound healing efficacy, we used 129 strain Oxttm1Wsy/J mice genetically unable to produce oxytocin (*oxt-KO*). With the same skin biopsy technique detailed previously, the strain 129 *oxt-KO* mice at 8-10 weeks of age were compared with their *wild type* (*oxt-WT*) littermates at six days after biopsy surgery. As predicted, skin wound repair was worsened in the absence of oxytocin. In particular, oxytocin was required for accelerated skin closure after eating *L. reuteri* (Figs. 6a and 6b). O*xt-KO* mice fed *L. reuteri* exhibited tissue repair deficits including delayed re-epithelialization (Fig. 6b), delayed collagen and fibrinogenesis (Fig. 6c), and increased accumulations of neutrophils (Fig. 6d), showing features characteristic of delayed wound repair [48] and dysregulated host immunity [49], when compared with their littermate *oxt-WT* mice. Delayed wound healing in oxytocin-deficient animals was also accompanied by significant increases in mast cells (Fig. S5a) and IL17+ macrophages (Fig. S5b), with decreased Foxp3+ cells (Fig. 6e), indicating an abnormally persistent immune reaction.

A requirement for oxytocin in *L. reuteri*-induced repair was also demonstrated in side-by-side comparisons between oxt-KO mice fed *L. reuteri* compared with Oxt-KO that did not receive *L. reuteri* in their drinking water. The oxt-KO animals did not experience significant differences in their wound healing parameters while drinking *L. reuteri*, when examined at 6 days after the skin biopsy procedure (Male: Oxt-KO+*L. reuteri* (n = 8) vs Oxt-KO (n = 7), wound closure difference p = 0.4557; Female: Oxt-KO+*L. reuteri* (n = 7) vs Oxt-KO (n = 7), wound closure difference p = 0.1014). In order to test the wound microenvironment's ability to receive direct oxytocin signals, we examined the wounds for oxytocin receptor expression with immunohistochemistry. Cells expressing oxytocin receptor, some of which were histomorphologically consistent with lymphocytes, were present within the wound sites of wild type mice consuming *L. reuteri* (Fig. S5c). Interestingly, deficits in tissue injury repair after skin biopsy were significantly improved within oxt-KO mice after only three days of intraperitoneal treatment with exogenous oxytocin throughout the entire recovery period (Fig. S6).

### Oxytocin-potent CD4^+^CD45RB^lo^ CD25^+^ Treg immune cells convey transplantable wound healing capacity

**Figure 7:**
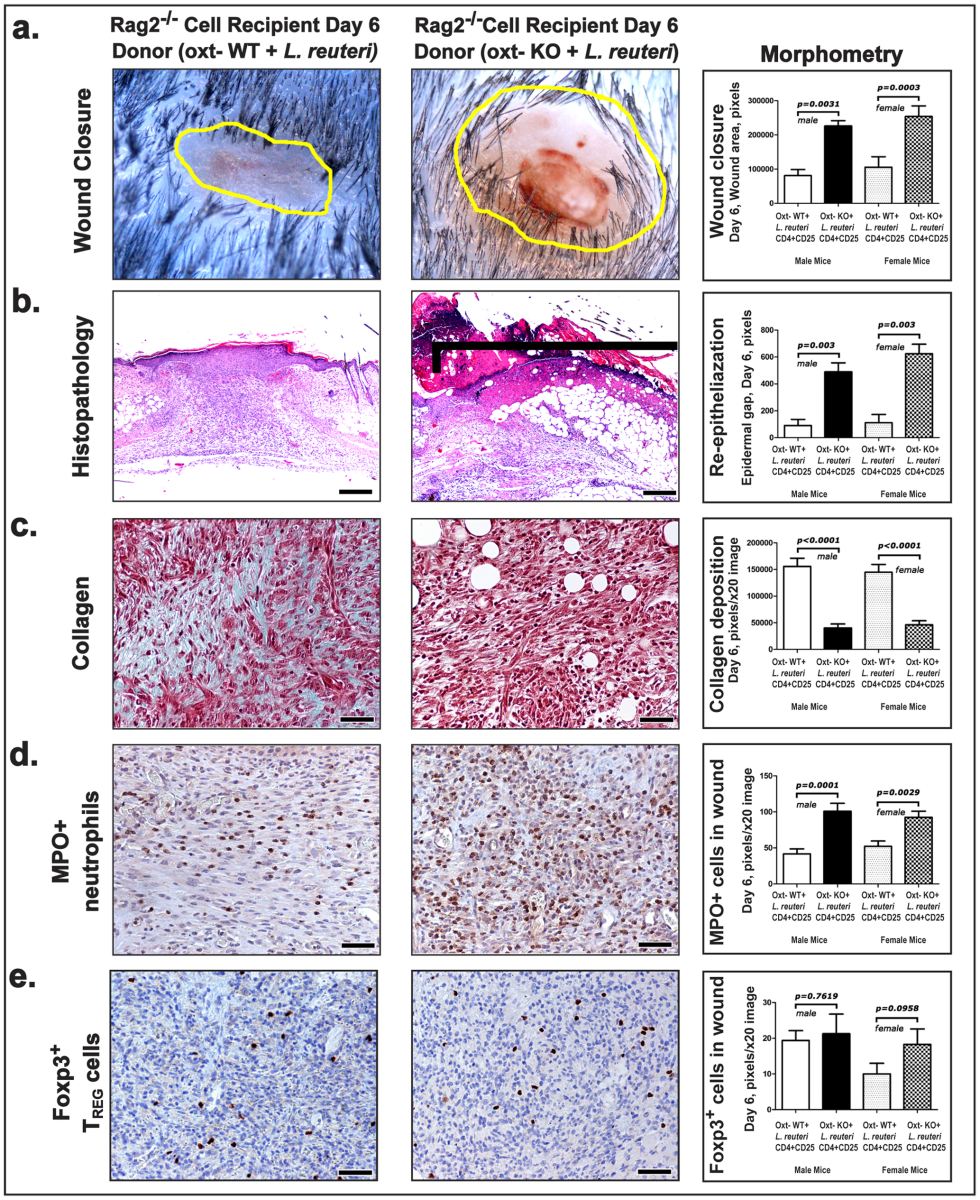
The oxytocin-dependent effect of *L. reuteri* is mediated by CD4+CD45RBloCD25+ Tregs. (a) Transferring CD4+CD45RBloCD25+ regulatory T (Treg) cells from oxytocin-potent *L. reuteri*-fed donor mice is sufficient to recapitulate the beneficial effects of probiotic consumption in the closure of cutaneous biopsy defects in Rag2–/– recipient mice. By contrast, Rag2 mice that got these same cells from *L. reuteri*-fed oxytocin-deficient donors failed to benefit, and instead presented large wounds at 6 days post- wounding. Whereas wounds of recipient mice of CD4+CD45RBloCD25+ cells of wild-type mice had accelerated wound healing, the recipients of oxytocin-deficient Tregs showed histopathological features of delayed wound healing, including significantly (b) delayed re-epithelialization, (c) decreased collagen deposition, (d) increased numbers of neutrophils, (e) and decreased regulatory T-cell in their wound counts. (b) Hematoxylin and Eosin. (c) Masson's Trichrome. (d and e) Immunohistochemistry; Diaminobenzidine chromogen, Hematoxylin counterstain. Scale bars: (b) =  250 µm; (c), (d) and (e) =  50 µm.

It was previously shown that oxytocin serves to up-regulate CD25 expression in thymic and peripheral lymphocytes [17], [22]–[23], [50]. Knowing that oxytocin up-regulates CD25 expression, and having shown that transplanted Treg cells were sufficient to convey wound repair into C57BL/6 Rag mice, we tested whether CD4^+^CD25^+^ Treg cells alone are sufficient for oxytocin-mediated wound repair effects [51]. To achieve this goal we applied a well-established adoptive transfer paradigm using highly purified CD4^+^CD45RB^lo^CD25^+^ Tregs collected from mesenteric lymph nodes and spleen [38]–[40]. To isolate the effects of these lymphocytes on host physiology, 4X10^3^ cells were injected intraperitoneally into 129 strain *Rag2*-deficient (*Rag2-KO*) animals otherwise lacking functional lymphocytes [39]. We found that transfer of CD4^+^CD45RB^lo^CD25^+^ Tregs alone from oxytocin-potent (oxt-WT) mice receiving *L. reuteri* led to significantly more rapid wound healing progression in recipient *Rag2-KO* animals that were themselves drinking untreated water (Fig. 7a). Strain 129 *Rag2-KO* recipient mice exhibited tissue repair improvements including more complete re-epithelialization (Fig. 7b), accelerated collagen deposition (Fig. 7c), and decreased accumulations of neutrophils (Fig. 7d), and comparable levels of skin-associated Foxp3+ cells (Fig. 7e) when receiving cells from mice that consumed *L. reuteri*.

**Figure 8:**
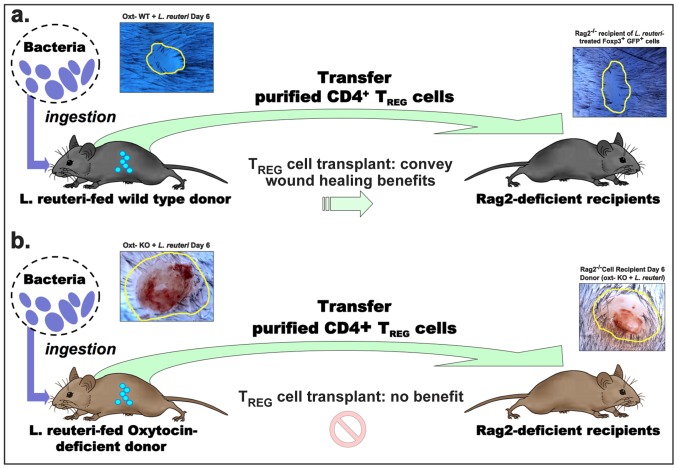
*L. reuteri* primes T regulatory cells via an oxytocin-dependent mechanism for enhanced wound healing. Wild type mice fed *L. reuteri* in their drinking water exhibit enhanced wound healing over untreated controls. This effect is entirely transferable with Foxp3+ Tregs primed in a mouse host feeding on *L. reuteri*. In the absence of oxytocin, feeding mice with *L. reuteri* provides no wound healing benefit, and the CD4+CD25+CD45Rblo Tregs from these animals are unable to convey wound-healing advantages.

In contrast, lymphocytes from *oxt-KO* mice eating *L. reuteri* imparted delayed wound repair processes (Figs. 7a and 7b) with delayed collagen deposition (Figs. 7c) and increased inflammatory component (Fig. 7d) resembling aspects of neoplasia-associated inflammation [52]; indeed, other studies show CD4^+^CD45RB^lo^CD25^+^ Tregs from immune-competent donor animals were similarly sufficient to inhibit inflammation-associated carcinogenesis in certain tissues [39]. Taken together, microbe-induced oxytocin fosters a host environment conducive to induction of CD4^+^CD45RB^lo^CD25^+^ Tregs imparting homeostasis [53]–[54]. We conclude that physiological events triggered by ingestion of lactic acid bacteria arise from microbe-host interactions by an oxytocin-mediated mechanism serving to activate lymphocytes that subsequently improve wound repair efficiency (Fig. 8).

## Discussion

We discovered that animals consuming purified lactic acid bacteria from human milk healed their wounds more than twice as fast as control animals. The associations of symbiotic gut organisms with various physiological and disease processes has been the focus of intense research over recent decades [1]–[4]. The present study, however, is the first to show that transient introduction of microbes into the gut microbiota, in this case with *L. reuteri*, bestows a distant extra-intestinal tissue, the integument, with accelerated wound healing capacity.

Successful healing of skin wounds requires a tightly orchestrated series of events with complex cell signaling cascades coordinating several fundamental biological processes [31]–[32]. The time course of these events was previously well-characterized in C57BL/6 mice, which have been extensively used in these types of investigations [48], [55]. With this strain of mice, we probed critical events of the stepwise experimental wound healing process *in situ* at 3, 6 and 12 days post-wounding. The processes of re-epithelialization and granulation tissue formation with collagen deposition and successive influx of different subsets of immune cells match the classical wound healing timeline previously described in C57BL/6 mice [33], [48], [56]. The *L. reuteri*-treated mice did not diverge from the histomorphological features of this paradigm. However, microscopically-recognized classical wound healing steps occurred much earlier in *L. reuteri*-treated mice when compared to controls. The *L. reuteri-*induced compression of wound healing events led to complete re-epithelialization at day 6, which coincided with increased granulation tissue maturity typified by quiescent fibroblasts, increased collagen deposition, absence of neutrophils, and increased lymphocytes including Foxp3+ regulatory T-cells. Superficially, this left the appearance of minimal scar formation in the excision wound site in animals treated with *L. reuteri* in their drinking water.

The increase in Foxp3+ Tregs in *L. reuteri*-fed mice matches previous reports showing that lactic acid bacteria stimulate the development of regulatory-T cells *in vitro* as well as *in vivo* [57]–[59]. It is reasonable that Foxp3+ Tregs contribute to the rapid clearance of neutrophils in the wound environment [60]–[61]. Although neutrophils are essential immediately after injury, their further persistence delays wound healing [62]–[63]. Our experiments demonstrate that depletion of CD25^+^ cells abolishes the beneficial effect of *L. reuteri* in wound healing. We also show that the depletion of the pro-inflammatory cytokine IL-17A, a well-known target of Treg anti-inflammatory activity [44]–[45], contributes to more rapid wound closure [64]. Finally, adoptive transfer of CD4^+^CD25^+^ Tregs is sufficient for the wound healing benefit. These results support a causal role for regulatory T-cells in integumentary wound healing. Further studies are required to clarify whether specific priming of Treg cells with certain bacteria is required for this beneficial effect and how the molecular profile of these cells changes under different exposure conditions.

An interesting corollary to accelerated healing properties exhibited in the present study is that feeding *L. reuteri* in mice also induces an anagenic shift in their hair follicles [16]. A recent study suggests that skin wounds made during the anagen stage show accelerated re-epithelialization [64]. Together, these studies reveal that reduction in inflammatory cell infiltration is the most probable explanation for rapid wound closure [64]–[65]. Thus, the *L. reuteri* anagen-inducing and anti-inflammatory properties may overlap as explanations of the accelerated wound healing phenomenon.

We observed an up-regulation of the neuropeptide hormone oxytocin following administration of purified *L. reuteri* organisms in drinking water. This finding, if translatable to humans, may be particularly important given the expanding roles of oxytocin in CNS-related functions, particularly social memory and attachment, sexual and maternal behavior, aggression, human bonding and trust, learning ability, anxiety, feeding behavior and pain cognition [17]–[19], [46], [66]–[68]. During early life, oxytocin regulates neurotransmitter gamma-aminobutyric acid (GABA) signaling in the central nervous system [69] providing a favorable mood reward [26] for social and gluttary gratification. Emerging data connect oxytocin with metabolism [20]–[21] and the immune system [22]–[24], providing satisfying evidence for its role as a global regulatory hormone. This finding not only connects microbial symbionts with social and immune fitness in evolutionary success, but also highlights enormous medical utility of microbe-induced oxytocin.

Because wound healing ability serves as a barometer for systemic health and fitness [31], we tested the role of oxytocin by studying wound healing capabilities of knockout mice at 6 days after wound induction. Delayed wound healing in oxytocin-deficient animals accompanied significant increases in neutrophils, mast cells, and Il-17^+^ macrophages, with decreased Foxp3^+^ cells, indicating an abnormally persistent reaction. Both wild type and oxytocin-deficient animals carried occasional oxytocin-receptor positive immune cells. Likewise, oxytocin-deficient mice had Foxp3+ cells within their convalescing wounds, indicating that Treg cells are present but unable to offer *L. reuteri*-induced wound repair assistance in the absence of oxytocin.

Oxytocin-mediated lymphocyte activation due to oral *L. reuteri* treatment could be an indirect or perhaps direct effect, since lymphocytes have been reported to possess a cell membrane receptor for oxytocin [17], [22]. In our studies using immune-competent mice, cells of skin innate immunity, such as neutrophils and mast cells, were reduced within wounds at the 6-day time-point as a result of the *L. reuteri*-induced, Treg-associated, oxytocin-mediated effect. This result matches a previous study in which the increased capacity of oral tolerance in mice to heal their skin wounds correlated with decreased numbers of mast cells and neutrophils in their wounds. That study concluded that excessively robust neutrophilic and mast cell immune responses may have slowed-down the healing process [51].

Our results suggest the presence of a multi-directional microbiome-gut-brain-immune system network in which oxytocin plays a pivotal role [14]–[15], [25]–[26], [70]–[71]. The vagus nerve serves as a bidirectional communication channel between the gut and the brain [26]–[29], and is required for gut-brain signaling initiated by *L rhamnosus* upregulating central GABA receptor expression [26]. The full *L. reuteri*-induced up-regulation of oxytocin and rapid wound repair required an intact vagus nerve as suggested by the unimproved wound healing phenotypes in vagotomized mice.

Our previous work and that of many others revealed that CD4^+^ cells are a basic cellular carrier of microbially-triggered signals that shape the immune system to restore homeostasis in inflammatory-associated pathologies, cancer, and reproductive health [6] [9], [42], [45], [70], [72]–[75]. We discovered that the *L. reuteri* benefit was transferable using CD4^+^ cell subsets alone, as long as those cells were from oxytocin-potent donors. In our previous studies we showed that homeostatic properties of CD4^+^ cells reside in a sub-population of CD4^+^ CD45RB^lo^ CD25^+^ T lymphocytes (Tregs) [76]–[78]. Transplanted CD4^+^ CD45RB^lo^ CD25^+^ Tregs alone completely recapitulated the *L. reuteri*-induced, oxytocin-dependent phenomenon in Rag-deficient recipient mice. These novel associations with oxytocin may link the few previously published reports suggesting that upon birth oxytocin simultaneously up-regulates IFN-γ and CD25 expression culminating in robust yet tightly regulated immunity for establishing self *vs.* non-self [22]–[23], [50].

The association between Foxp3^+^ Tregs and oxytocin may involve a cellular trafficking role, based on lowered numbers of Foxp3^+^ cells in the wound sites of oxy-KO mice fed *L. reuteri* compared to wild type also fed *L. reuteri*. Notably, the increase in Foxp3^+^ Tregs in wounds after administration of *L. reuteri* in wild type mice and in wounds of mice receiving *L. reuteri*-primed Foxp3+ Tregs also indicate possible proliferative or stimulatory aspects for oxytocin. In *Rag2-KO* mice, still capable of producing oxytocin, adoptively transferred CD4^+^FoxP3^+^ Tregs from either oxy-KO or wild type donors fed *L. reuteri* will traffic to wound sites, but with performance deficits in oxy-KO cells. That exogenous oxytocin administration restores the ability of oxy-KO mice to heal their wounds supports the idea that trafficking and stimulation of Tregs in wound sites is potentially a direct consequence of circulating oxytocin levels. Extensive *in vivo* and *in vitro* characterizations of Treg biology under these various conditions is currently underway in our laboratory.

From an evolutionary perspective, we assert that lactic acid bacteria co-evolved with mammals partially by exploiting oxytocin to optimize reproductive fitness. As a regulator of immune competence, oxytocin may provide selective advantage to hosts in conditions where dedicating energy to immune function is vital: namely child-rearing and living in social or kin groups. Elevated plasma oxytocin levels in our separate studies correlated with improved maternal care and neonatal survival rates in mother C57BL/6 mice consuming *L. reuteri* (data not shown). Oxytocin has been inversely linked with post-partum depression and maternal neglect in human females [79], highlighting substantial maternal and infant gains during symbiotic microbial interactions. Benefits of this microbial synergy may extend beyond maternal-infant bonding, to social superiority with oxytocin enhancing cooperation within social groups while promoting aggression towards competitors [80]. These inter-related roles for oxytocin may impact a natural selection process favoring complex social organizations required for evolutionary success.

The combination of oxytocin with sex-linked physical, reproductive, and immune responses could have selective advantages in mammalian hosts – providing social functionality concomitant with improved immune function for survival in groups. In fact, in humans, specific SNPs in the 3′ untranslated region of the oxytocin receptor gene correlate with prosocial behavior, suggesting that modulation of these hormones at the levels of transcription and translation can have profound influence on these phenotypes [81]. Because microbes such as *L. reuteri* seem to modulate the expression of oxytocin, there is a clear role in co-evolution of microbial symbionts with their mammalian hosts for enhancing reproductive fitness and consequently also microbial proliferation.

Different impacts of *L. reuteri* on wound-healing according to gender may arise from the overlay of oxytocin with a sex-specific hormonal milieu. Female mice fed *L. reuteri* experience enhanced wound-healing over their male counterparts, suggesting that this microbe co-opts the endocrine environment of its host for effect. Interestingly, male wild type mice exhibited greater improvements in wound healing compared with male oxytocin-deficient mice, versus the female wild type mice compared to their oxytocin-deficient female counterparts. This observation suggests that females have alternative compensatory mechanisms in the absence of oxytocin in preparing the immune system for the wound healing process. We noted in our previous studies [16] that female mice showed a more robust response in terms of skin pH and fur luster than do male mice. This phenotype likely reflects the ability of sex-steroid hormones to influence immunological processes in a sexually dimorphic manner. Generally, females mount more robust humoral and cell-mediated immunity than males; in the absence of oxytocin, females may benefit from these inherent advantages in immunity over males in wound healing [82]. The loss of a key hormonal regulator of the immune response for males results in significant challenges during wound repair and likely in other cases requiring tight regulation of cell-mediated immunity.

Our findings also raise diverse benefits for orally administered microbes to lessen impairments during normal aging, imparting health resiliency typical of much younger individuals. Wound healing capacity directly translates to nearly every aspect of traditional health and medicine [20], [83]. We identify key mechanisms uniting social support networks with improved injury repair in healthful longevity [47]. Humans have cultivated and consumed similar food-grade organisms in fermented beverages and active yogurts for thousands of years, supporting a low-risk, high-impact population-based approach. This microbe-endocrine-immune linkage has the potential to reduce hospitalizations, improve healing, lower risk for certain cancers, and enhance healthful aging.

## Experimental Procedures

### Animals

C57BL/6 wild type (wt), oxytocin-deficient WT and knockout (KO) B6;129S-Oxttm1Wsy/J mice (purchased initially from Jackson labs; Bar Harbor, ME), C57BL/6 Foxp3^EGFP^ which co-express eGFP and the regulatory T cell-specific transcription factor Foxp3 (Jackson labs), and 129SvEv Rag2-ko mice (Taconic Farms; Germantown, NY) were housed and handled in Association for Assessment and Accreditation of Laboratory Animal Care (AAALAC)-accredited facilities using techniques and diets including Lactobacillus reuteri as specifically approved by Massachusetts Institute of Technology's Committee on Animal Care (CAC) (MIT CAC protocol # 0912-090-15 and 0909-090-12). The MIT CAC (IACUC) specifically approved the studies as well as the housing and handling of these animals. Mice were bred in-house to achieve experimental groups. The B6;129S-Oxttm1Wsy/J mice were back-crossed eight generations to the 129 strain background prior to using for adoptive transfer experiments with Rag2-KO mice. Each experiment included 5-15 animals per group with one or two replications (total N = 10–30 mice examined per group) unless otherwise specified.

Mice underwent a standardized 2.0mm cutaneous biopsy procedure [76], while under general inhalant anesthesia, and with pre- and post-operative buprenorphine analgesia. Tissues for analyses were collected upon necropsy at specified time points. For the initial studies, C57BL/6 *wt* mice, receiving either *L. reuteri* treatment or regular water, underwent tissue biopsy at approximately six months of age, in order to mimic wound repair in middle-aged humans subjects, and were examined at three, six, or twelve days post-biopsy. For subsequent experiments, oxt-KO mice and their oxt-WT littermates entered experiments at 6-8 weeks of age. These mice underwent tissue biopsy at approximately 10–12 weeks of age, and were finally euthanized with CO_2_ and examined by morphometry as described below.

### Special microbial treatments for animals

Mice were fed standard rodent chow (MRH 3000; Purina Labs, St Louis MO). Subsets of animals were supplemented orally with a strain of *Lactobacillus reuteri* [ATCC-PTA- 6475], originally isolated from human milk, and subsequently cultivated as described elsewhere [16], [34], using a supply dosage of 3.5×10^5^ organisms/mouse/day continuously in drinking water. For the initial studies, C57BL/6 wt mice at 6 months of age received *L. reuteri* as above, for at least two weeks prior to skin biopsy. For subsequent studies, *oxt-KO* and their littermate *oxt-WT* mice began drinking *L. reuteri* organisms, as above, starting at 6–8 wks of age, and then underwent biopsy approximately 2–3 weeks later at 10–12 weeks of age. Drinking water was replaced twice weekly to minimize variability in microbial exposure levels. Sham control mice received an *E. coli* non-pathogenic lab strain of K12 at the same dosage of 3.5X10^5^ organisms/mouse/day in drinking water, as specified. Other control animals received regular drinking water.

### Measurement of plasma oxytocin levels

Whole blood was collected terminally by cardiac puncture under general anesthesia to obtain plasma. Blood was collected into pre-chilled 5 ml EDTA tubes with 250 KIU of apoprotinin, and refrigerated until processing. Plasma was isolated by centrifugation at 1800 g, 15 minutes, 4°C, and then stored in aliquots at −70°C. Plasma was then tested commercially (*AniLytics*,. Inc., Gaithersburg, MD). Euthanasia for mice was performed mid-day for all subjects (n = 10 per group) to minimize variability due to Circadian rhythms.

### Vagotomy procedure

C57BL/6 wt mice (n =  8 per group) underwent vagotomy at 8 weeks of age. The vagotomy procedure used gaseous isoflourane anesthesia. Once a surgical plane of anesthesia is assured by toe pinch, the surgical site was prepared, and the stomach and lower esophagus were visualized by upper midline laparotomy. After visualization, both vagal trunks including all neural and connective tissue surrounding the esophagus and below the diaphragm was removed to transect all small vagal branches. Sham surgery did all procedures except to truncate the vagus nerve. All animals received pro-operative and post-operative buprenorphine analgesia. At least a two-wk recovery period was allowed prior to initiation of probiotics and skin biopsy surgery. [26]

### Supplementation with exogenous oxytocin

Exogenous oxytocin was used to independently determine impact on wound healing. Mice (n = 6 per group) were injected intraperitoneally every 4–6 hours with 15 units/mouse of oxytocin (Bimeda-MTC Animal Health Inc., Cambridge, Canada) suspended in Lactated Ringers solution (LRS). Sham treated mice received LRS solution only on the same dosing schedule. Treatments began 24–48 hours prior to skin biopsy, and continued throughout until euthanasia three days later.

### Skin biopsy procedure

Mice underwent a 2.0mm cutaneous skin punch biopsy procedure as described elsewhere[30], [35]while under general inhalant anesthesia, and with pre- and post-operative buprenorphine analgesia. Tissues for analyses were collected upon necropsy at specified time points of three days, six days or 12 days after biopsy.

### Excision (biopsy) wound area and epithelial closure assessments

Formalin-fixed, routinely-processed, paraffinized, flat wounded skin tissues were used for wound area measurements before being embedded in paraffin blocks. Direct microscopy with a Nikon eclipse 50i microscope and a Nikon DS-5 M-L1 digital camera was used to examine and photograph wounds in paraffinized gross skin specimens. The wound areas were measured in images using the ImageJ image processing and analysis program (NIH, Bethesda, MD). Results were recorded as image pixels. In addition, histological sections showing the maximum wound epidermal gap were used to evaluate re-epithelialization. The epidermal gap was measured in low power x2 images. Results were recorded as pixels.

### Assessment of wound healing rates

To calculate the wound healing rate constant, log-transformed wound area was fit using regression analyses. Slopes predicting the rate constants were compared with an interaction model with proc reg in SAS (alpha  =  0.05) [84]–[85].

### Depletion of CD25^+^ cells

Mice were treated with anti-CD25 antibody (clone PC-61; Bio- X-cell, West Lebanon, NH) at 150 ug per mouse intraperitoneally 3X weekly for 12 weeks. Treated mice were compared to mice receiving sham isotype antibody alone. Depletion of CD25^+^ cells was confirmed by undetectably low fractions of CD25+ cells in spleens of mice treated with anti-CD25 antibody compared to sham-treated controls using flow cytometry. Depletion was also confirmed by absence of Foxp3^+^ cells in mesenteric lymph nodes using immunohistochemistry on fixed tissues.

### Neutralization of IL17A

Mice were treated with anti-IL17A antibody (Bio-X-cell, West Lebanon, NH) at 150 ug per mouse intraperitoneally 3X weekly for 12 weeks. Treated mice were compared to mice receiving sham isotype antibody alone.

### Adoptive transfer of T cells into recipient mice

CD4^+^ lymphocytes were isolated from C57BL/6 Foxp3^EGFP^ which co-express eGFP and the regulatory T cell-specific transcription factor Foxp3 using magnetic beads (Dynal) for initial sorting, and then further sorted by flow cytometry to isolate gfp+ cells. In addition, CD4^+^ Treg lymphocytes isolated from 129 strain *wild type or oxytocin-deficient* mice used magnetic beads (Dynal) for initial sorting, and then were further sorted by hi-speed flow cytometry (MoFlow2) to obtain purified populations of CD4^+^ cells or CD4^+^CD45RB^lo^CD25^+^ lymphocytes determined to be ∼96% pure as previously described elsewhere. In both models, Rag2^−/−^ recipient mice were then injected intraperitoneally with 4X10^6^ cells. All recipient animals were provided regular drinking water without supplementary *L. reuteri* and underwent skin biopsy at three weeks after cell transfer.

### Histopathology and immunohistochemistry

Formalin-fixed wounded skin and mesenteric lymph node tissues were embedded in paraffin, cut at 4–5 µm, and stained with hematoxylin and eosin (HE). Step sectioning was applied in order to reach the proximity of the mid-wound level in skin samples. Then, multiple serial sections were taken from each sample for HE, Masson's trichrome and Toluidine blue stains and immunohistochemistry. Sections showing the maximum wound epidermal gap were used to evaluate re-epithelialization. The epidermal gap was measured in low power x2 images. Results were recorded as pixels. Immunohistochemistry (IHC) and morphometric assessment were as previously described[76]. IHC-positive immune cells and green-stained fibrous tissue with Masson's trichrome stain were counted in x20 images and results were recorded as number of cells or number of green collagen pixels per image. Primary antibodies for IHC included rabbit polyclonal antibodies for myeloperoxidase (ThermoFisher Scientific/Lab Vision, Fremont, CA), IL-17 (Santa Cruz Biotechnology, Inc., Santa Cruz, CA), cleaved caspase-3 (Cell Signaling, Beverly, MA) and oxytocin-receptor (Abcam, Cambridge, UK), rabbit monoclonal antibodies for C-kit and Ki-67 (Cell Marque, Rocklin, CA) and a rat monoclonal antibody for Foxp3 (eBioscience, Inc., San Diego, CA) detection. Primary antibody binding was detected with goat anti-rabbit polymer HRP (ZytoChem Plus, Berlin, Germany) or biotinylated goat-anti rat IgG (Serotec, Oxford, UK). Heat-induced antigen retrieval was performed with citrate buffer, pH 6, for myeloperoxidase, caspase-3 and oxytocin-receptor, with EDTA buffer, pH 8, for C-kit and foxp3 detection or with CC1 epitope retrieval solution (Ventana Medical Systems, Inc., Tucson, AZ) for ki-67 and IL-17. The ImageJ image processing and analysis program (NIH, Bethesda, MD) was used for all quantitative histomorphometry assessments.

### Statistical analyses

For all statistical analyses the Mann-Whitney U test (Graphpad Prism version 4.0 for windows, Graph-Pad software, San Diego, CA, USA) was used. Effects were considered to be significant at p<0.05. The y-axis of bar graphs stands for the mean±SEM of counts indicated in each graph.

Word count  =  5,808 (without title, abstract, formatted references, acknowledgements, or figure captions)

## Supporting Information

Figure S1 ***L. reuteri*-induced accelerated wound repair exhibits key histopathological features at day 6.** Control mice at day 6 post-wounding have incomplete re-epithelialization (arrow-head marks the leading edge of re-epithelialization). An area of the granulation tissue in the wound bed (inset) is shown bellow in higher magnification. The immature granulation tissue is loose, edematous and has emerging vessels and many activated (plump) fibroblasts (arrow), abundant neutrophils (black arrow-head ) and macrophages (white arrow-head). In contrast, the wounds of *L. reuteri*-treated mice at the same time-point show complete re-epithelialization and the granulation tissue in the wound bed is more mature. The boxed area is shown bellow in higher magnification. The mature granulation tissue has absent neutrophils and a chronic inflammatory component (lymphocytes-black arrow-head), elongated fibroblasts (black arrow-head) and early deposition of collagen fibers (white arrow-head). Hematoxylin and Eosin. Scale bars: upper panels low magnification = 250 µm; high magnifications of the inset areas (lower panels) = 25 µm.
(TIF)

Figure S2 **Cellular proliferation in wounds at 6 days post-wounding.** Proliferation features reflect the more advanced healing stage conferred by interconnected roles of (a) *L. reuteri*, (b) oxytocin and (c) regulatory T-cells. A large number of ki-67+ epidermal cells locate at the migrating edges of the epidermis in the open wounds of control experimental groups. The newly formed epidermis sealing the wounds of mice as a result of treatment has less proliferating cells. In the wound bed, early-stages of wound healing are characterized by the high proliferating activity of neutrophils (N) and fibroblasts (F). The more advanced stage of wound healing in animals receiving treatments is characterized by less proliferating fibroblasts. Immunohistochemistry: Diaminobenzidine chromogen, Hematoxylin counterstain. Scale bars = 50 µm.
(TIF)

Figure S3 **Apoptosis in wounds at 6 days post-wounding.** Similarly to proliferation, apoptosis in wounds (probed by Caspase-3-specific immunohistochemistry) also reflects the more advanced healing stage due to interrelated activity of (a) *L. reuteri*, (b) oxytocin and (c) regulatory T-cells. Although there are no caspase 3+ cells in the epidermis, increased numbers of apoptotic fibroblasts are seen in the granulation tissue bed only in the untreated experimental groups showing accelerated wound healing. Immunohistochemistry: Diaminobenzidine chromogen, Hematoxylin counterstain. Scale bars = 50 µm.
(TIF)

Figure S4 **Vagotomy impairs the *L. reuteri*-induced acceleration of wound repair.** (a) Vagotomy abolishes the *L. reuteri*-induced systemic elevation of oxytocin indicating that the signaling responsible for up-regulating the expression of oxytocin is transmitted via the vagus nerve. In contrast to control mice undergoing sham-surgery, vagotomized C57BL/6 mice fail to fully benefit from *L. reuteri* consumption and show (b) larger wounds and (c) only partial re-epithelialization.
(TIF)

Figure S5 **(a) Oxytocin-receptor immune cells exist in the skin wounds of mice.** Upper panel: postpartum mouse uterus serves as a positive control whereby epithelial cells of the endometrium and smooth muscle cells of the myometrium are positively stained. The negative control of the stain is shown in the upper right. Oxytocin-receptor-positive immune cells are evident within the granulation tissue of wounds. Arrows point to positively labeled immune cells, which are morhologically consistent with lymphocytes. Oxytocin-receptor-specific immunohistochemistry: Diaminobenzidine chromogen, Hematoxylin counterstain. Scale bars = 25 µm. **(b and c) Key pro-inflammatory cells fail to subside in oxytocin-deficient mouse skin wounds.** At day 6 post-wounding the number of (b) Toluidine-blue stained, c-kit-positive mast cells and (c) IL-17 positive macrophages is significantly higher in oxytocin-KO mice consuming *L. reuteri* compared to their oxytocin-WT counterparts. Upper panel of (b): Toluidine Blue stain, Lower panel of (b) and (c) c-kit- and IL-17-specific immonihistochemistry respectively (Diaminobenzidine chromogen, Hematoxylin counterstain). Toluidine Blue scale bars = 50 µm. C-kit and IL-17 IHC scale bars = 25 µm.
(TIF)

Figure S6 **Exogenous administration of oxytocin restores normal wound healing in oxytocin-deficient mice.** Oxytocin-KO mice treated with exogenous oxytocin by intraperitoneal injections have smaller wounds and increased re-epithelialization when examined at 3 days post-wounding compared to the sham Lactated Ringers solution-injected controls. Histopathology: Hematoxylin and Eosin. Scale bars = 250 µm.
(TIF)
