# Supplementary figures and images for: Microbial Symbionts Accelerate Wound Healing via the Neuropeptide Hormone Oxytocin
Source: PLoS One. 2013 Oct 30;8(10):e78898. doi: 10.1371/journal.pone.0078898 (PMC3813596; doi:10.1371/journal.pone.0078898)

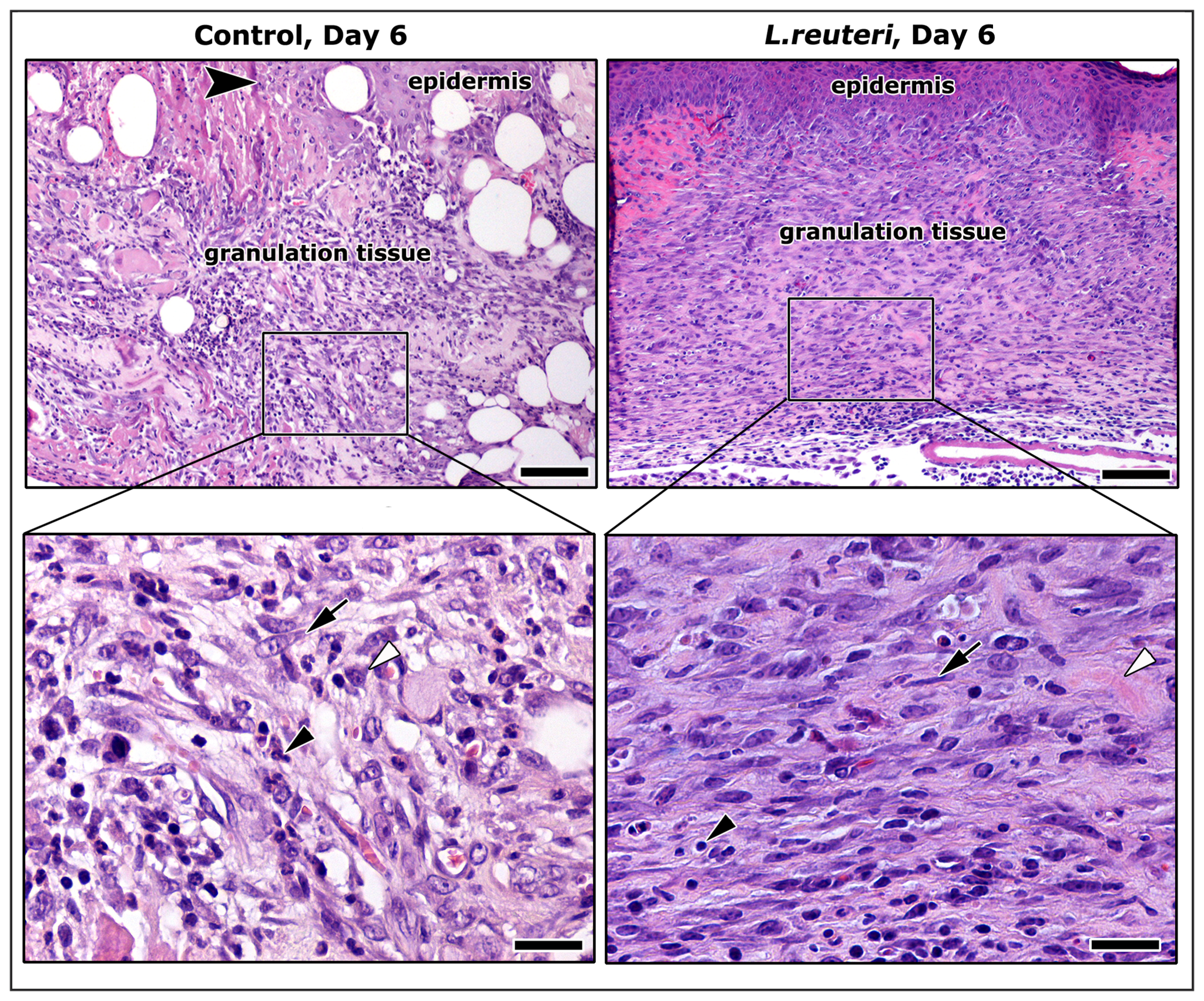

Supplement: Figure S1 — L. reuteri-induced accelerated wound repair exhibits key histopathological features at day 6. Control mice at day 6 post-wounding have incomplete re-epithelialization (arrow-head marks the leading edge of re-epithelialization). An area of the granulation tissue in the wound bed (inset) is shown bellow in higher magnification. The immature granulation tissue is loose, edematous and has emerging vessels and many activated (plump) fibroblasts (arrow), abundant neutrophils (black arrow-head ) and macrophages (white arrow-head). In contrast, the wounds of L. reuteri-treated mice at the same time-point show complete re-epithelialization and the granulation tissue in the wound bed is more mature. The boxed area is shown bellow in higher magnification. The mature granulation tissue has absent neutrophils and a chronic inflammatory component (lymphocytes-black arrow-head), elongated fibroblasts (black arrow-head) and early deposition of collagen fibers (white arrow-head). Hematoxylin and Eosin. Scale bars: upper panels low magnification = 250 µm; high magnifications of the inset areas (lower panels) = 25 µm. (TIF) [file pone.0078898.s001.tif]

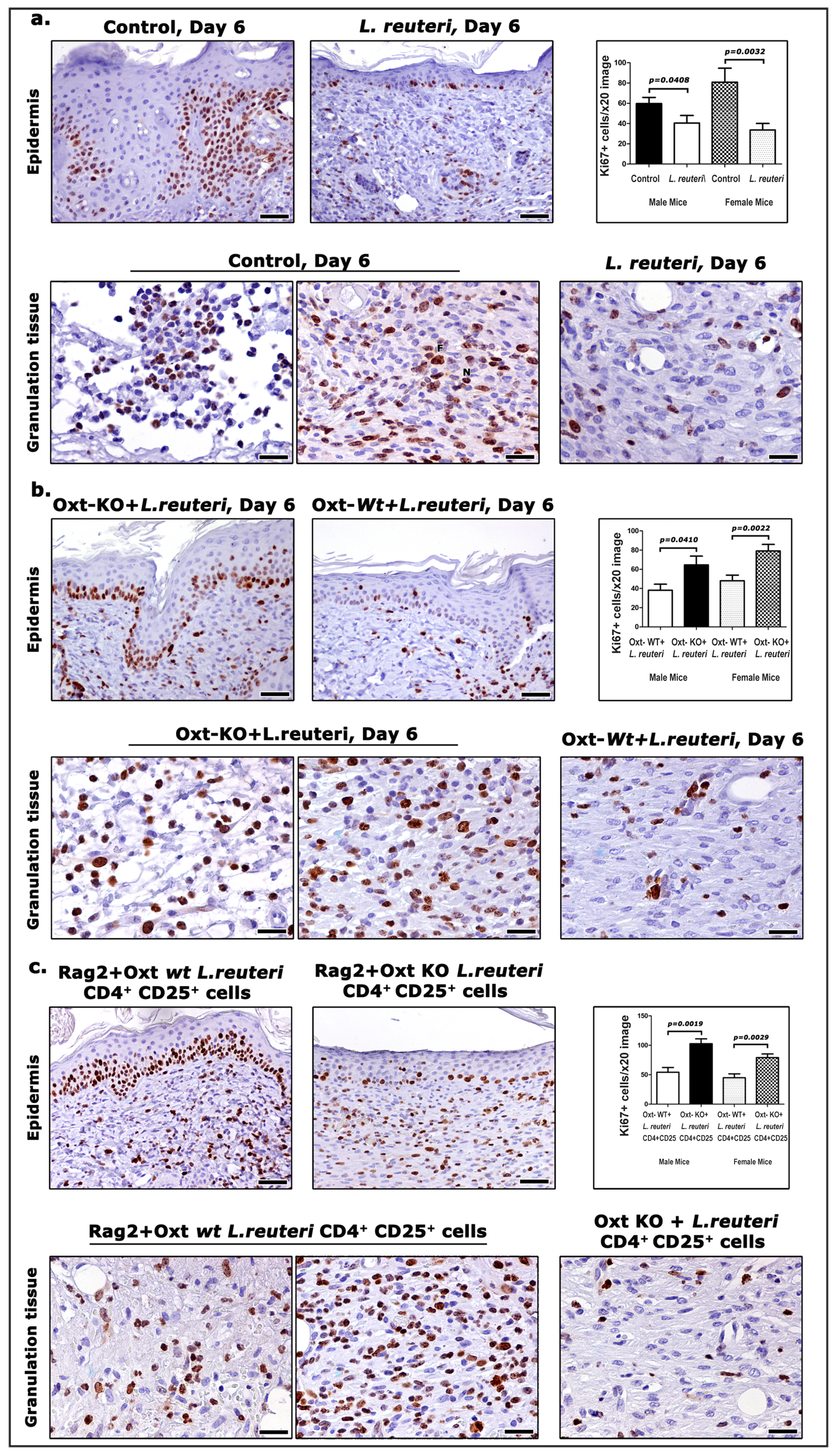

Supplement: Figure S2 — Cellular proliferation in wounds at 6 days post-wounding. Proliferation features reflect the more advanced healing stage conferred by interconnected roles of (a) L. reuteri, (b) oxytocin and (c) regulatory T-cells. A large number of ki-67+ epidermal cells locate at the migrating edges of the epidermis in the open wounds of control experimental groups. The newly formed epidermis sealing the wounds of mice as a result of treatment has less proliferating cells. In the wound bed, early-stages of wound healing are characterized by the high proliferating activity of neutrophils (N) and fibroblasts (F). The more advanced stage of wound healing in animals receiving treatments is characterized by less proliferating fibroblasts. Immunohistochemistry: Diaminobenzidine chromogen, Hematoxylin counterstain. Scale bars = 50 µm. (TIF) [file pone.0078898.s002.tif]

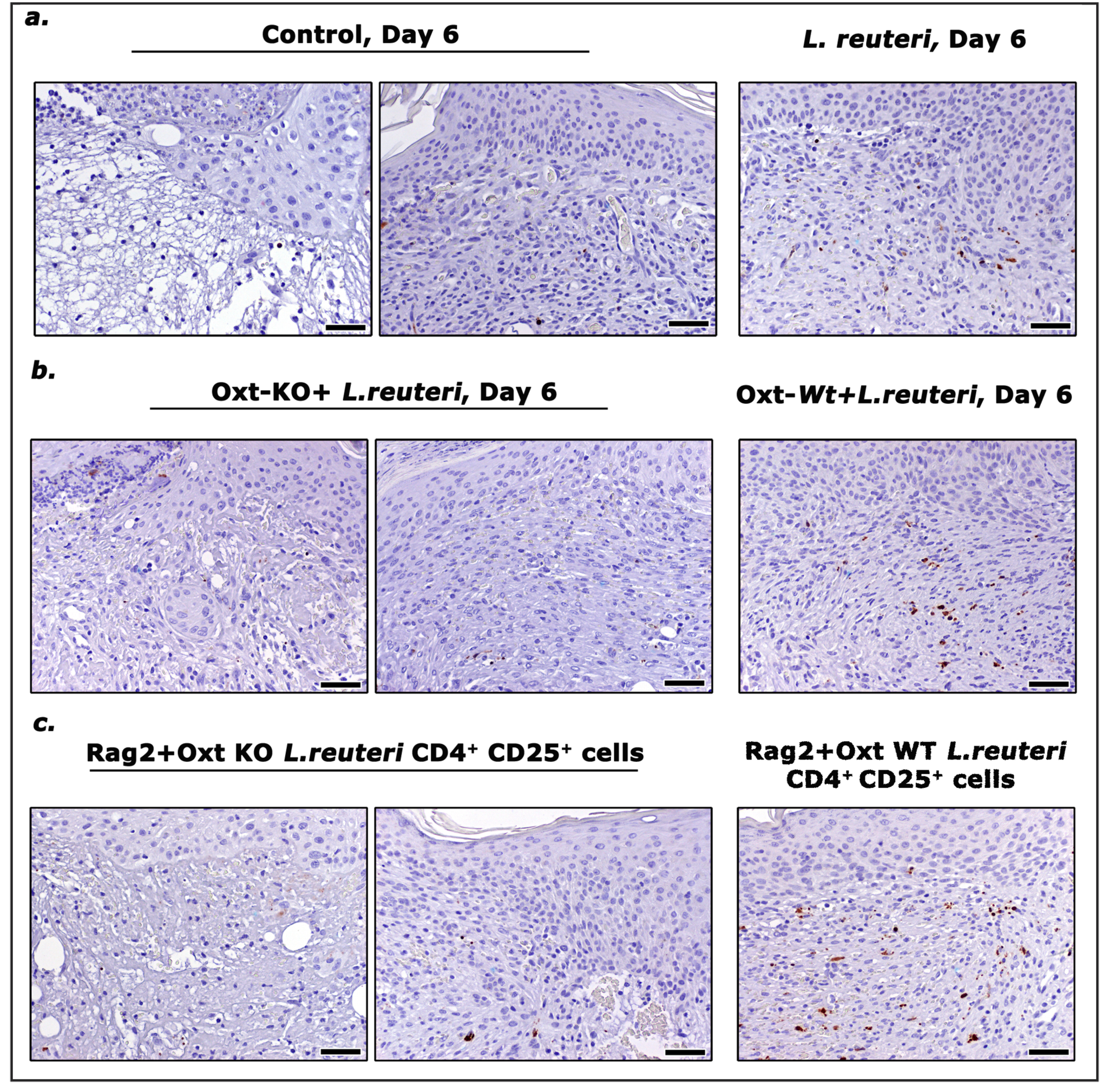

Supplement: Figure S3 — Apoptosis in wounds at 6 days post-wounding. Similarly to proliferation, apoptosis in wounds (probed by Caspase-3-specific immunohistochemistry) also reflects the more advanced healing stage due to interrelated activity of (a) L. reuteri, (b) oxytocin and (c) regulatory T-cells. Although there are no caspase 3+ cells in the epidermis, increased numbers of apoptotic fibroblasts are seen in the granulation tissue bed only in the untreated experimental groups showing accelerated wound healing. Immunohistochemistry: Diaminobenzidine chromogen, Hematoxylin counterstain. Scale bars = 50 µm. (TIF) [file pone.0078898.s003.tif]

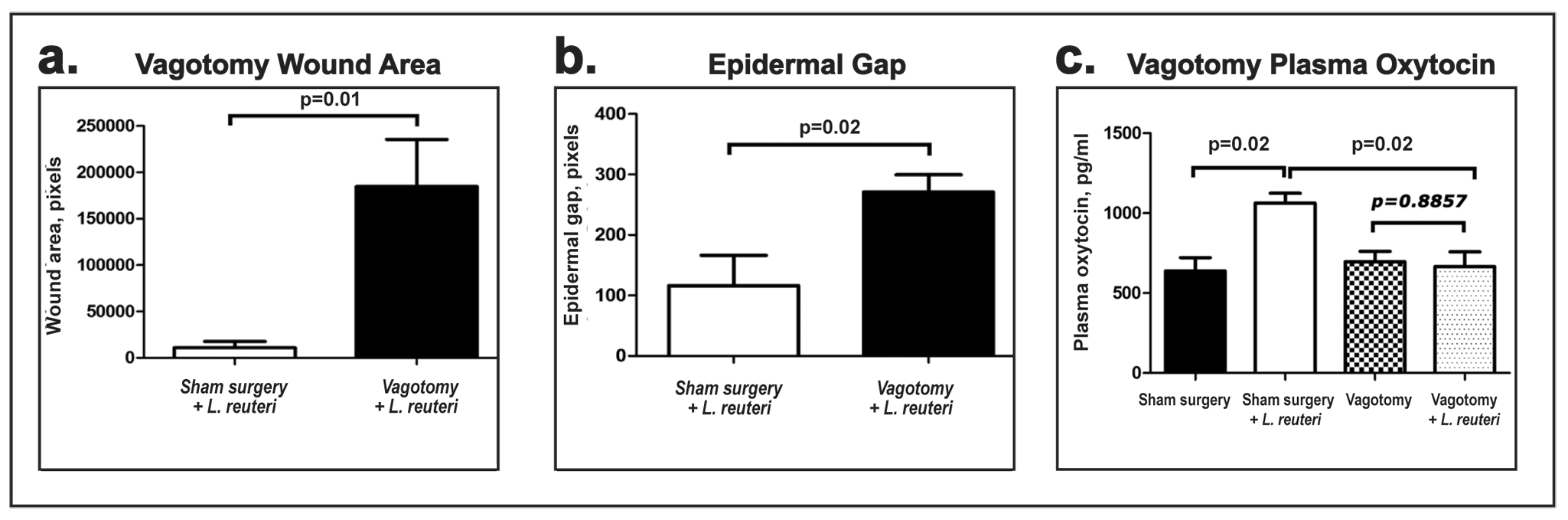

Supplement: Figure S4 — Vagotomy impairs the L. reuteri-induced acceleration of wound repair. (a) Vagotomy abolishes the L. reuteri-induced systemic elevation of oxytocin indicating that the signaling responsible for up-regulating the expression of oxytocin is transmitted via the vagus nerve. In contrast to control mice undergoing sham-surgery, vagotomized C57BL/6 mice fail to fully benefit from L. reuteri consumption and show (b) larger wounds and (c) only partial re-epithelialization. (TIF) [file pone.0078898.s004.tif]

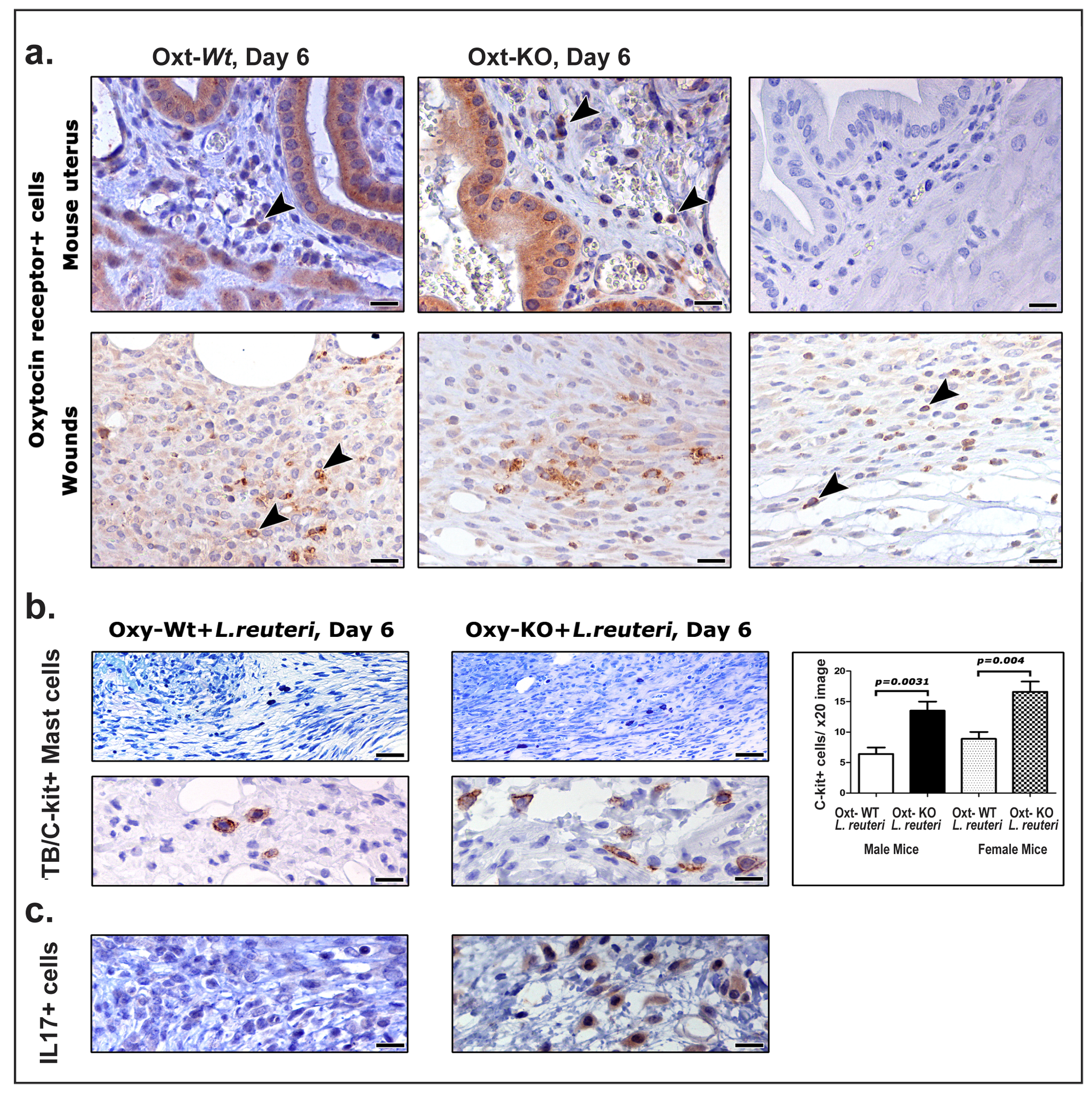

Supplement: Figure S5 — (a) Oxytocin-receptor immune cells exist in the skin wounds of mice. Upper panel: postpartum mouse uterus serves as a positive control whereby epithelial cells of the endometrium and smooth muscle cells of the myometrium are positively stained. The negative control of the stain is shown in the upper right. Oxytocin-receptor-positive immune cells are evident within the granulation tissue of wounds. Arrows point to positively labeled immune cells, which are morhologically consistent with lymphocytes. Oxytocin-receptor-specific immunohistochemistry: Diaminobenzidine chromogen, Hematoxylin counterstain. Scale bars = 25 µm. (b and c) Key pro-inflammatory cells fail to subside in oxytocin-deficient mouse skin wounds. At day 6 post-wounding the number of (b) Toluidine-blue stained, c-kit-positive mast cells and (c) IL-17 positive macrophages is significantly higher in oxytocin-KO mice consuming L. reuteri compared to their oxytocin-WT counterparts. Upper panel of (b): Toluidine Blue stain, Lower panel of (b) and (c) c-kit- and IL-17-specific immonihistochemistry respectively (Diaminobenzidine chromogen, Hematoxylin counterstain). Toluidine Blue scale bars = 50 µm. C-kit and IL-17 IHC scale bars = 25 µm. (TIF) [file pone.0078898.s005.tif]

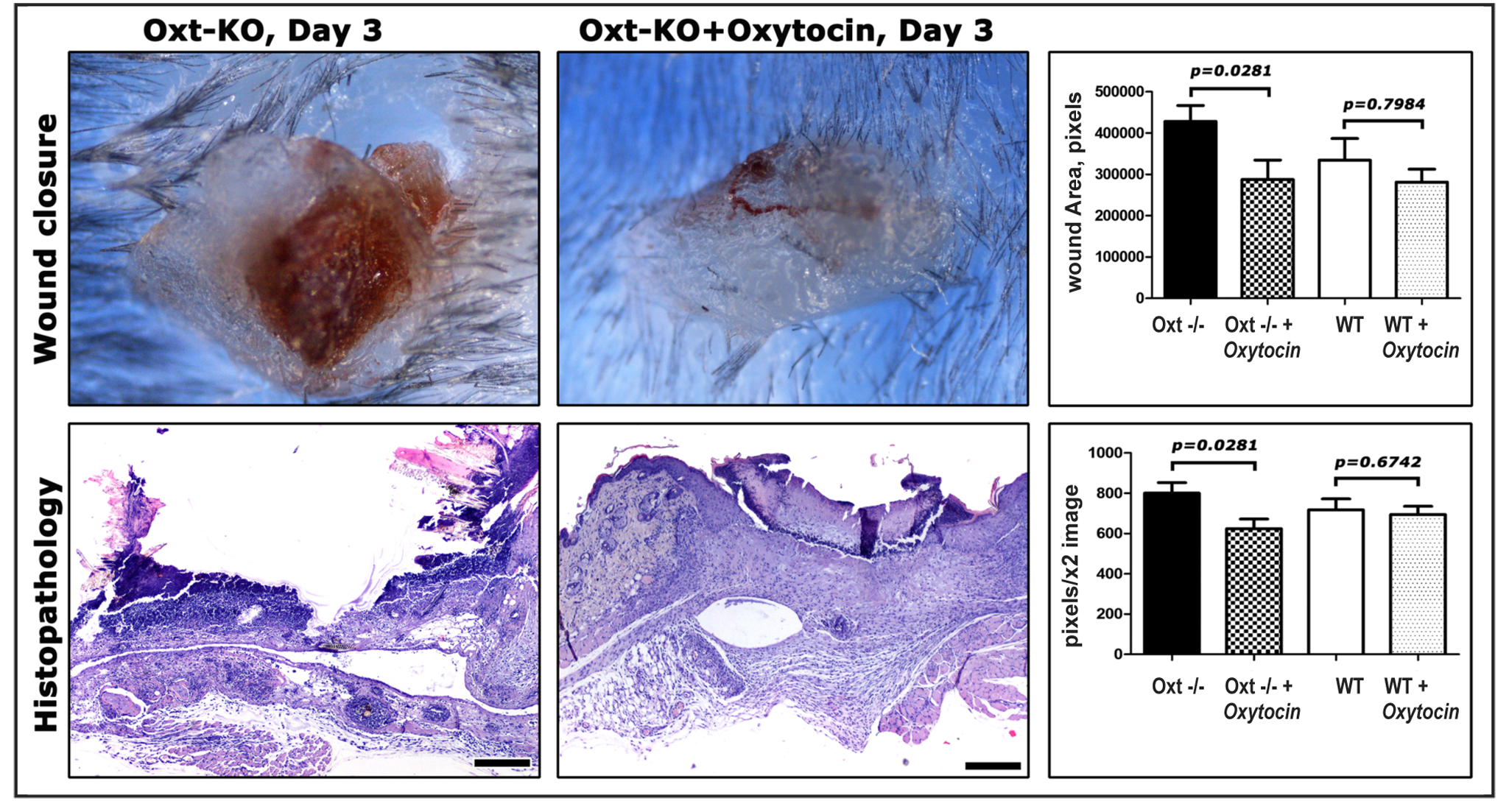

Supplement: Figure S6 — Exogenous administration of oxytocin restores normal wound healing in oxytocin-deficient mice. Oxytocin-KO mice treated with exogenous oxytocin by intraperitoneal injections have smaller wounds and increased re-epithelialization when examined at 3 days post-wounding compared to the sham Lactated Ringers solution-injected controls. Histopathology: Hematoxylin and Eosin. Scale bars = 250 µm. (TIF) [file pone.0078898.s006.tif]
